# Deep anomaly detection of seizures with paired stereoelectroencephalography and video recordings

**DOI:** 10.1038/s41598-021-86891-y

**Published:** 2021-04-05

**Authors:** Michael L. Martini, Aly A. Valliani, Claire Sun, Anthony B. Costa, Shan Zhao, Fedor Panov, Saadi Ghatan, Kanaka Rajan, Eric Karl Oermann

**Affiliations:** 1 Department of Neurosurgery, Icahn School of Medicine at Mount Sinai, New York, NY 10029 USA; 2 Department of Neurosciences, Friedman Brain Institute, Icahn School of Medicine at Mount Sinai, One Gustave Levy Place, New York, NY 10029 USA; 3 Department of Anesthesiology, Icahn School of Medicine At Mount Sinai, New York, NY 10029 USA; 4 Department of Neurosurgery, New York University Langone Medical Center, New York University, Skirball, Suite 8S, 530 First Avenue, New York, NY 10016 USA; 5 Department of Radiology, New York University Langone Medical Center, New York, NY 10016 USA; 6 NYU Center for Data Science, New York University, New York, NY 10011 USA

**Keywords:** Machine learning, Epilepsy

## Abstract

Real-time seizure detection is a resource intensive process as it requires continuous monitoring of patients on stereoelectroencephalography. This study improves real-time seizure detection in drug resistant epilepsy (DRE) patients by developing patient-specific deep learning models that utilize a novel self-supervised dynamic thresholding approach. Deep neural networks were constructed on over 2000 h of high-resolution, multichannel SEEG and video recordings from 14 DRE patients. Consensus labels from a panel of epileptologists were used to evaluate model efficacy. Self-supervised dynamic thresholding exhibited improvements in positive predictive value (PPV; difference: 39.0%; 95% CI 4.5–73.5%; Wilcoxon–Mann–Whitney test; N = 14; p = 0.03) with similar sensitivity (difference: 14.3%; 95% CI − 21.7 to 50.3%; Wilcoxon–Mann–Whitney test; N = 14; p = 0.42) compared to static thresholds. In some models, training on as little as 10 min of SEEG data yielded robust detection. Cross-testing experiments reduced PPV (difference: 56.5%; 95% CI 25.8–87.3%; Wilcoxon–Mann–Whitney test; N = 14; p = 0.002), while multimodal detection significantly improved sensitivity (difference: 25.0%; 95% CI 0.2–49.9%; Wilcoxon–Mann–Whitney test; N = 14; p < 0.05). Self-supervised dynamic thresholding improved the efficacy of real-time seizure predictions. Multimodal models demonstrated potential to improve detection. These findings are promising for future deployment in epilepsy monitoring units to enable real-time seizure detection without annotated data and only minimal training time in individual patients.

## Introduction

Epilepsy is among the most common neurological disorders worldwide with an estimated 5 million people diagnosed each year^1^. Epileptic seizures are characterized by pathological electrical activity in regions of the brain that manifest as functional disturbances that may be transient^2^. Although first-line treatment to control seizures consists of antiepileptic drugs, more than 30% of patients are pharmacoresistant and at high risk for premature mortality^3–5^. Stereoelectroencephalography (SEEG) is a method for localizing epileptogenic foci in patients with drug resistant epilepsy (DRE) involving placement of macroelectrode depth electrodes into the brain, followed by continuous monitoring in a specialized epilepsy monitoring unit (EMU)^6–8^. Epileptologists must quickly recognize abnormal SEEG waveforms, and EMU staff must monitor patients for signs of clinical seizures around the clock, making this is a highly time- and resource-intensive process.

Deep learning-based approaches are promising solutions to automated seizure detection, but they are not without limitations^9–13^. Previous studies have: (1) used algorithms engineered to classify previously recorded EEG sequences without a framework for real-time event detection, (2) required large training datasets, extensive annotations, and a pre-screening for artifacts to achieve adequate results, and (3) produced high false positive rates, commonly due to static thresholding methods applied in the decision function. This limits clinical utility, particularly in the context of large-scale data produced by continuous in-hospital recordings. Furthermore, acquiring large, annotated datasets and screening for artifacts is time- and cost-prohibitive which diminishes utility unless the pre-trained models are exceptionally well-generalizable. Given the variety of waveforms, dynamic noise, and other idiosyncrasies often present in patient recordings, seizure detection remains challenging.

**Figure 1:**
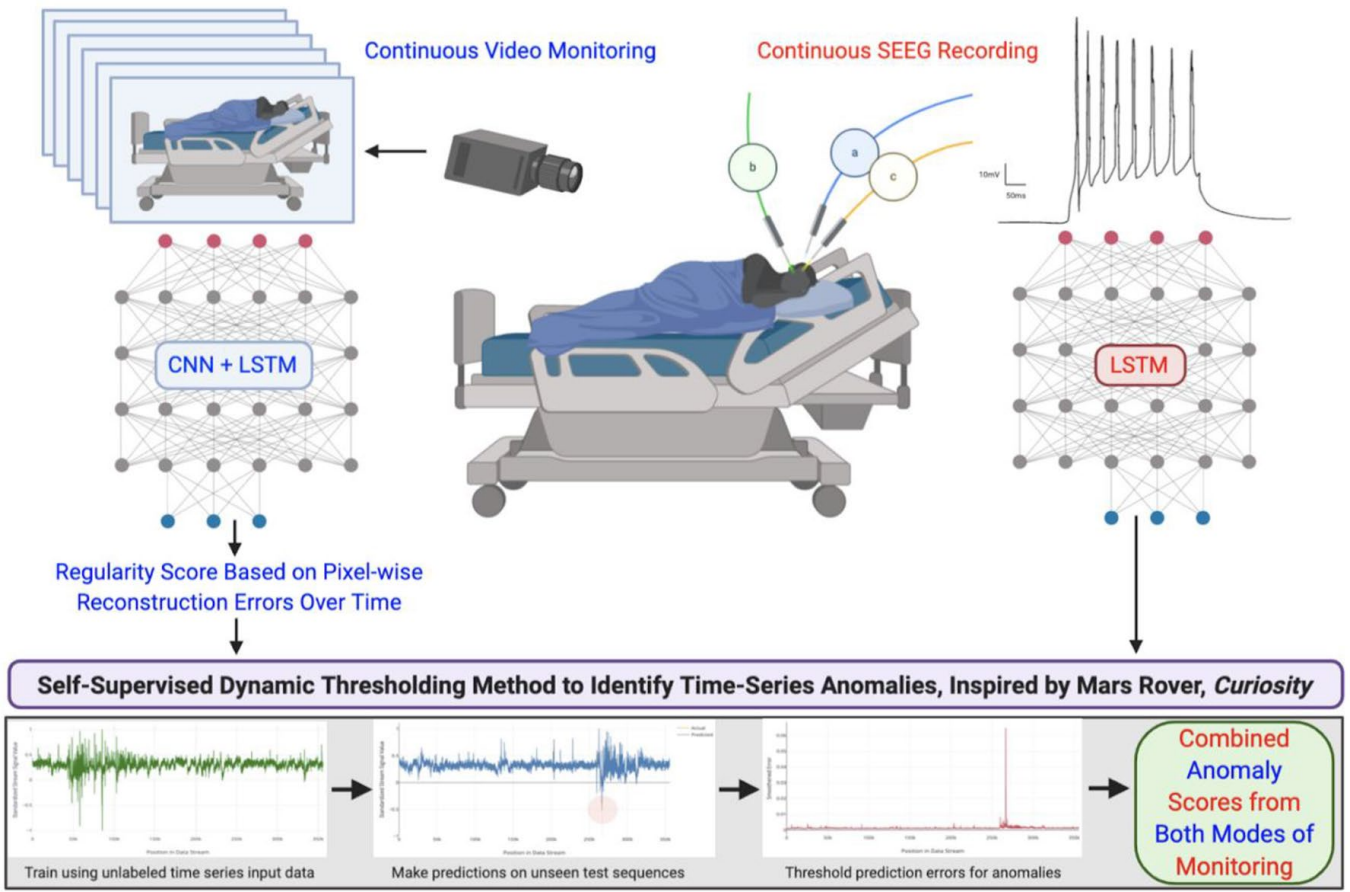
Overview of the workflow for continuous monitoring with video and SEEG and real-time analysis in the epilepsy monitoring unit. Patients with DRE receive continuous monitoring of their intracranial SEEG leads (red) and simultaneous video recording in their hospital beds (blue). A convolutional LSTM autoencoder (CNN + LSTM) was applied to the video recordings to calculate a regularity score for each frame over time. This regularity score time series and the SEEG time series (green sequence, bottom left) were then separately fed into an LSTM network to reconstruct their signals (blue sequence, bottom middle) and calculate a reconstruction error (red sequence, bottom right) which was then subjected to a self-supervised dynamically thresholding method to identify anomalous events in real-time.

We present our results from training individually tailored, self-supervised Long Short-Term Memory (LSTM) deep neural networks on continuous in-hospital multichannel SEEG and video recordings with no explicitly labeled data (Fig. 1). Here, we define seizure detection as the task of anomaly detection in high-dimensional sequences. A dynamic thresholding method, developed by NASA for use on the Mars Rover, was adapted to improve detection sensitivity and mitigate false positives, suggesting feasibility as a new, more dynamic paradigm for real-time anomaly detection in video and electroencephalographic data^14^.

**Figure 2:**
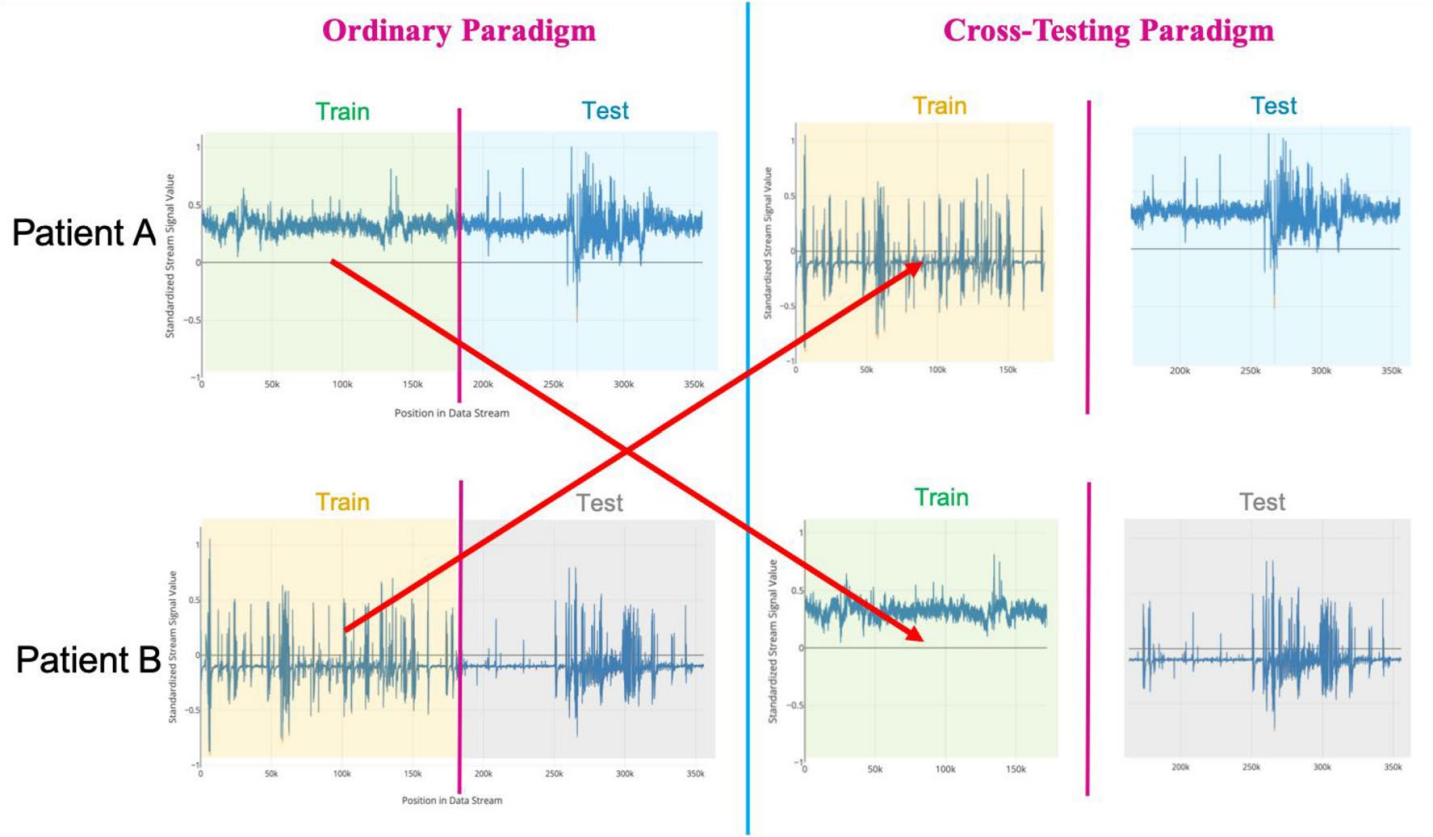
Design of crossover experiments to assess patient-specificity of models. LSTM models were trained on recordings from one patient and tested on recordings from another patient.

**Figure 3:**
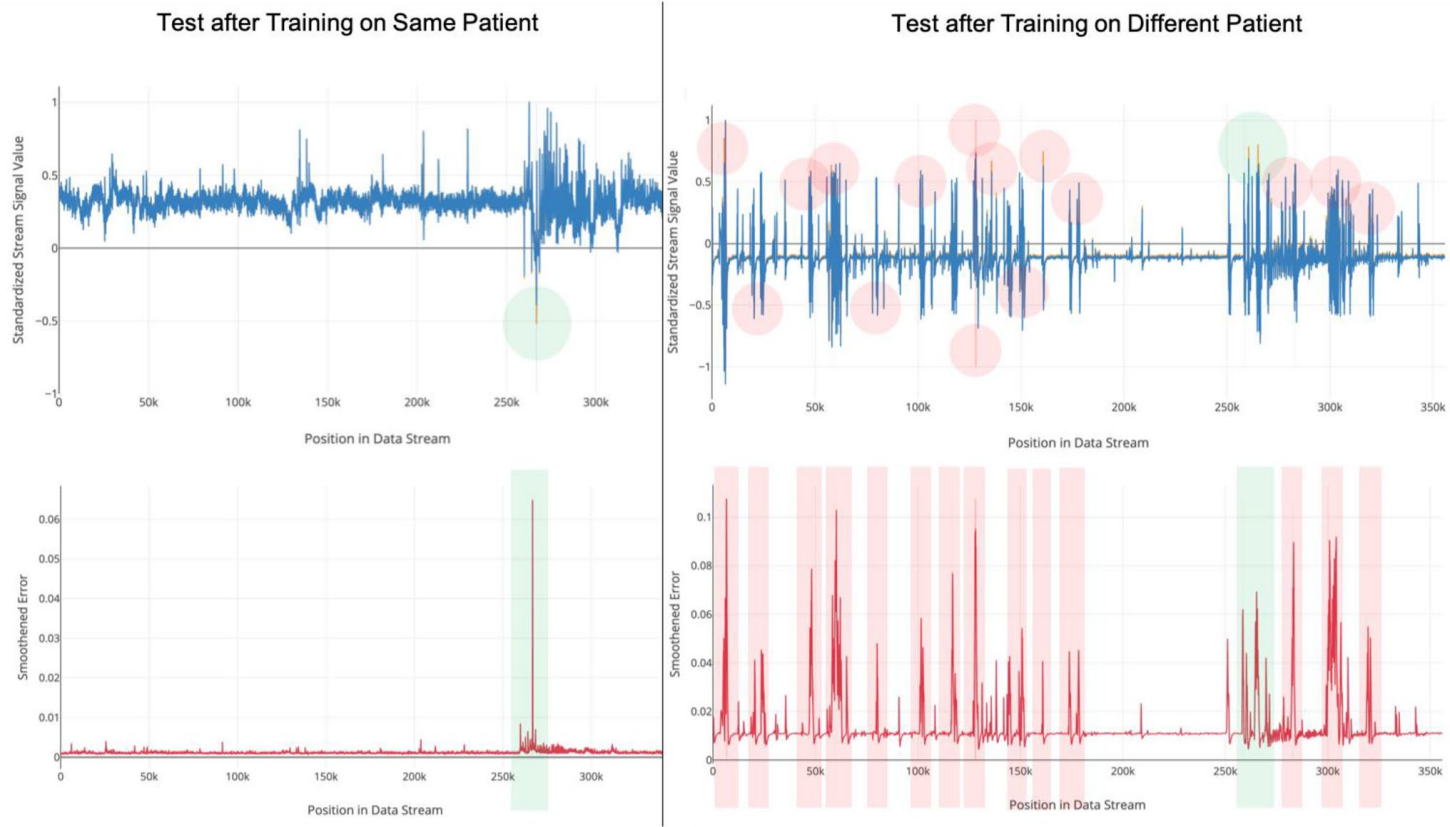
Crossover testing produces a large increase in the number of false positive results. This indicates that trained models are attuned to the unique electrical signal of a given patient. Green shading refers to prediction mismatches that correspond to correctly identified anomalies whereas red shading refers to prediction mismatches that correspond to false positives.

Concurrent SEEG and video signals, totaling over 2000 h across all patients and channels analyzed, were processed by adapting previously described methods (“Signal processing”)^15,16^. LSTMs and convolutional LSTM autoencoders were trained for each patient as described in “LSTM training and parameters”. Dynamic thresholding was compared to conventional static thresholding, crossover experiments (Figs. 2, 3) were performed to characterize models’ patient-specificity, and joint models incorporating SEEG and video detection were constructed to assess the added benefit of multimodal detection. Model outputs were compared to ground truth anomalous sequences agreed upon by three fellowship-trained epileptologists who were blinded to the results of the model. The positive predictive value (PPV), sensitivity, and F_1_ scores were compared between models. Mean absolute percent error and minimal duration of recording data to train each model were also noted.

## Results

### Dynamic vs. static thresholding

**Figure 4:**
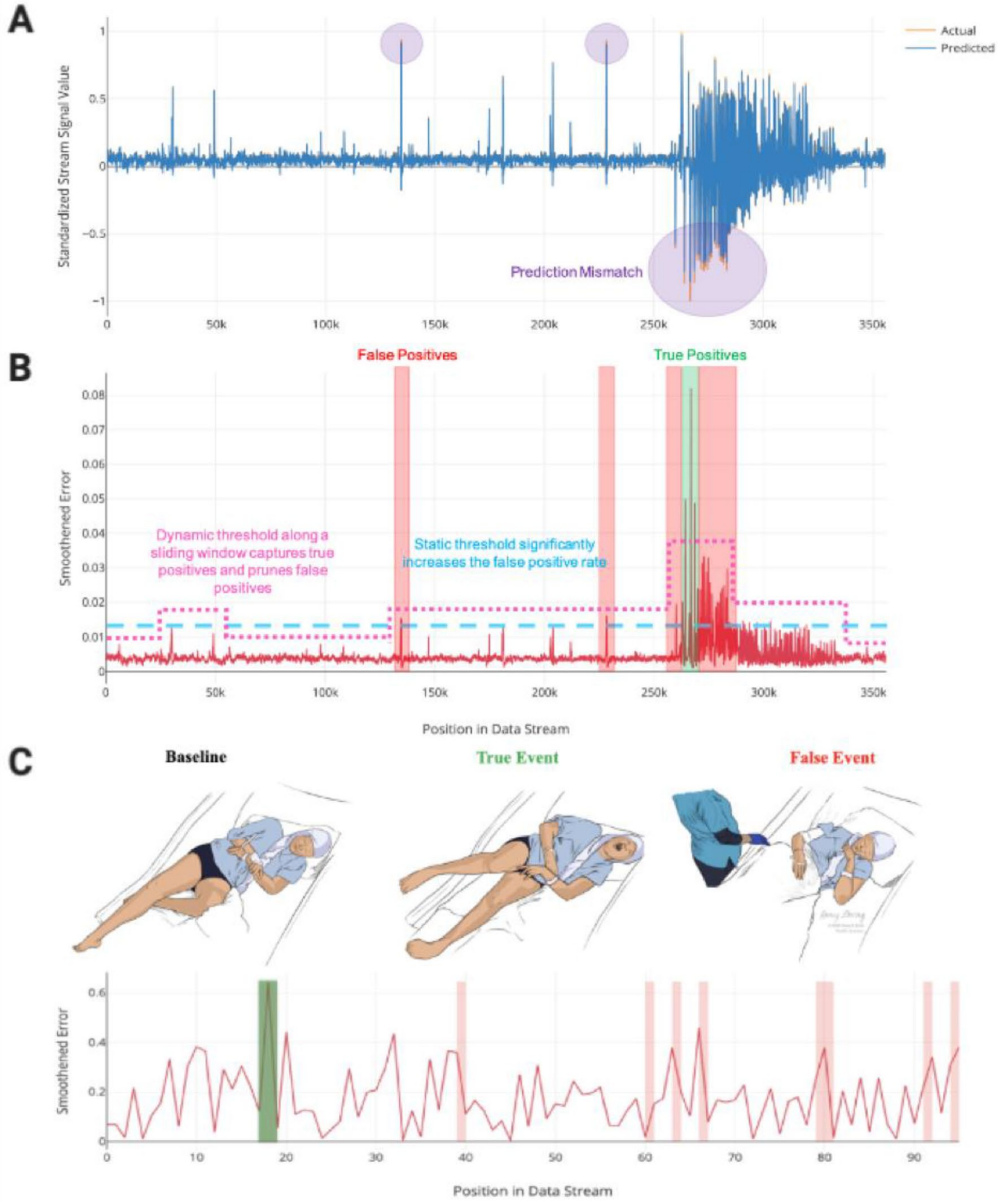
Self-supervised error thresholding for real-time detection of anomalies in SEEG and video data. An LSTM network is trained to predict the next window of values in the test time series sequence (**A**, blue). These values are compared to the actual values (**A**, orange), and a smoothed error is calculated for each value in the sequence (**B**, red sequence). Prediction mismatches (**A**, purple) manifest as higher errors. A self-supervised dynamic threshold (**B**, magenta line) enables effective local classification of true anomalous sequences (**B**, green bar) while omitting many of the false positives (**B**, red bars) that result from traditional static thresholding methods (**B**, blue line). Concurrently acquired video recordings for each patient were considerably noisier and signal reconstruction was not as robust, demonstrated by the higher reconstruction errors (**C**, red sequence). While video sequences captured all of the true seizure events in the study population (**C**, middle, green bar), they also captured several false positive events, such as nurse visits (**C**, far right, red bars).

The dynamic threshold with error pruning was compared to a baseline, fixed threshold, label-free anomaly detection approach. Inspection of the threshold demonstrated that the mathematical optimization in each window found localized levels that effectively categorized anomalies in real-time (Fig. 4A,B). Compared to static thresholding, dynamic thresholding did not improve sensitivity significantly (difference: 14.3%; 95% CI − 21.7 to 50.3%; Wilcoxon–Mann–Whitney test; N = 14; p = 0.42) but did significantly increase PPV (difference: 39.0%; 95% CI 4.5–73.5%; Wilcoxon–Mann–Whitney test; N = 14; p = 0.03). Additionally, F_1_ scores were significantly higher for the dynamic threshold (difference: 0.31; 95% CI 0.1–0.61; Wilcoxon–Mann–Whitney test; N = 14; p = 0.04).

### Crossover experiments

Crossover experiments assessed whether the learned features from each patient training model generalized to testing sequences derived from different DRE patients (Figs. 2, 3). With six distinct crossover combinations, anomaly detection sensitivity remained comparable to the non-crossover experiments (difference: 4.8%; 95% CI − 38.4 to 47.9%; Wilcoxon–Mann–Whitney test; N = 14; p = 0.82), but PPV (difference: 56.5%; 95% CI 25.8–87.3%; Wilcoxon–Mann–Whitney test; N = 14; p = 0.002) and F_1_ scores significantly declined (difference: 0.38; 95% CI 0.08–0.67; Wilcoxon–Mann–Whitney test; N = 14; p = 0.02). After training on continuous data from a given patient, testing the network on an unseen sequence derived from the same patient resulted in high fidelity of predicted sequences, with most of the prediction mismatches (Fig. 3, left, top, green circle) corresponding to true anomalies (Fig. 3, left, bottom, green bar). Testing this same model on an unseen, normalized sequence derived from a different patient produced considerably more prediction mismatches (Fig. 3, right, top, red and green circles), resulting in higher false positive rates (Fig. 3, right, bottom, red bars).

### Multimodal detection

Joint models incorporating self-supervised anomaly detection in video and SEEG recordings were constructed to determine the potential added benefit of multimodal detection (Fig. 4C). Multimodal detection significantly improved sensitivity (difference: 25.0%; 95% CI 0.2–49.9%; Wilcoxon–Mann–Whitney test; N = 14; p < 0.05) over dynamically thresholded SEEG recordings, but decreased PPV, though not significantly (difference: 21.3%; 95% CI − 10.3 to 52.9%; Wilcoxon–Mann–Whitney test; N = 14; p = 0.17). Relative to video detection alone, the combined workflow also improved the PPV (difference: 28.5%; 95% CI 4.6–52.4%; Wilcoxon–Mann–Whitney test; N = 14; p = 0.02) and F_1_ scores (difference: 0.22; 95% CI − 0.01 to 0.44; Wilcoxon–Mann–Whitney test; N = 14; p = 0.06).

## Discussion

The study is the first to implement a multimodal self-supervised deep learning workflow for intracranial seizure detection in DRE patients. While previous studies have used bedside recordings to classify hypermotor seizures, few have jointly evaluated video and electroencephalographic feeds to detect seizures^17,18^. This study provides a novel proof-of-concept in this arena by demonstrating the potential of self-supervised anomaly thresholding to improve the sensitivity and PPV of automated seizure detection on continuous multimodal recordings in real-time. Because error residuals in anomaly detection are often non-Gaussian, the nonparametric dynamic thresholding method for error classification used in this study overcomes a major limitation of prior studies using parametric thresholding methods which assumed a distribution that does not fit the residuals.

The pipeline presented in this work utilizes a LSTM network and a convolutional LSTM autoencoder to enable real-time detection of anomalous events in high-resolution SEEG and video data, respectively, making them valuable in a prospective setting. Models were trained on only 5–10 min of SEEG recordings which did not necessarily include a seizure event and labeled data was not required, thereby reducing time and cost of analysis. Crossover studies suggested the self-learned representations of SEEG recordings are patient-specific, which provides confidence in the ability of our algorithm to identify clinically relevant features given the diversity of signal properties between patients. Taken together, clinical translation of this work could personalize the care of patients and augment the workflow of staff in the EMU. By ingesting a few initial minutes of a patient’s recording, this pipeline would enable continuous long-term monitoring of ictal events and reduce frequent false alarms in the context of subtle environmental changes, which would otherwise be time intensive and cost prohibitive.

Earlier methods in inpatient epileptic seizure detection have traditionally relied more on constant monitoring of patient recordings by trained personnel. This is available in approximately 56–80% of EMUs, whereas automatic online EEG warning systems are present in only 15–19% of EMUs^19^. While clinical seizure semiology provides some critical information to help elucidate the zone of onset and propagation pathways, periictal behavioral assessments facilitate an even more comprehensive understanding of these details. Most algorithms for EEG-based seizure detection in clinical settings center around multiple-channel analyses rather than single-channel^19^. Following data acquisition by the electrodes, these systems typically employ a method for artifact rejection followed by an algorithm for event detection usually involving analysis of the electrographic changes during seizures in terms of amplitude, frequency, or rhythmicity. Methods for these analyses in previous algorithms for patient-specific seizure detection have included both linear and nonlinear time–frequency signal analysis techniques^20–22^. More recent studies focusing on automated seizure detection have relied on other machine learning techniques, including support vector machines, *k*-nearest neighbors, and convolutional neural networks, which require complete electroencephalograms before determining whether anomalies are present^23–25^. Such properties limit the application of these approaches primarily to retrospective data. Furthermore, unlike deep learning methods which learn the best features to implement to achieve optimal performance, these older methods require manual feature extraction and careful programming of the network to obtain acceptable results. Other work has focused on developing large pre-trained models with the goal of successful generalization to other patients^26^. Of note, there are several generalized, commercially-available seizure detection algorithms currently on the market, including Persyst-Reveal^27^, IdentEvent^28^, BESA^29^, and EpiScan^30^. The primary limitation of these methods, however, is that they may not generalize well to other patients given the wide variety of signal characteristics that may exist as a result of recording quality, patient disease and electrophysiological characteristics, or other uncontrollable factors. This, in turn, may limit clinical efficacy. In contrast, as described previously, the workflow presented in this study could be rapidly deployed in clinical settings to create patient-specific models with improved adaptability for prospective prediction.

### Limitations

This study’s limitations include using retrospective data for training and a relatively small patient cohort, which could introduce selection bias. While overfitting is always a concern in deep learning, we controlled for this by holding out data for validation for each patient and using early stopping criteria during model training (“LSTM training and parameters”). Additionally, although incorporating videos improved sensitivity, it also increased false positives. Developing more sophisticated tiered or weighted systems for escalating anomalies detected in concurrent multimodal recordings could reduce false positives in this workflow. Future work is underway to adapt these methods to a prospective, randomized format to confirm the utility of self-supervised dynamic thresholding for seizure detection in a clinical setting.

## Conclusions

Self-supervised dynamic thresholding of patient-specific models significantly improves the PPV of seizure detection in continuous SEEG recordings from DRE patients compared to traditional static thresholds. Incorporating concurrent video recordings into multimodal models significantly improved sensitivity, but reduced PPV, though not significantly. The characteristics of these models are promising for future deployment in clinical settings to improve the speed, precision, and cost-effectiveness of epilepsy monitoring, which may ultimately improve the safety profile of SEEG monitoring for our patients.

## Methods

### Study protocol

Patients with drug resistant epilepsy (DRE) at an academic medical center were retrospectively enrolled in the study. Subjects with significant progressive disorders or unstable medical conditions requiring acute intervention, those taking more than three concomitant antiepileptic drugs (AEDs) or with changes in AED regimen within 28 days, and patients with onset of epilepsy treatment less than two years prior to enrollment, were excluded from the study. In total, 14 consecutive DRE patients underwent surgical implantation of 10–18 multichannel SEEG leads from 2018–2019 as per standard hospital protocols (average: 15 leads, 147 channels) and subsequent in-hospital video and SEEG monitoring for 4–8 days (average: 6 days). Patients were 16–38 years old (average: 24.5 years), 57% were female, and 71.4% were taking AEDs during the recording period. All patients had recordings with at least one epileptiform event (Table 1). This study was approved by the Mount Sinai Health System Institutional Review Board (IRB). Informed consent was waived by the IRB with oversight from the Program for the Protection of Human Subjects Office. All methods were performed in accordance with their relevant guidelines and regulations.

**Table 1:** Characteristics of the patient population.

**Demographics**	
Age (mean ± SEM)	24.5 ± 2.0 years
Female (%)	8 (57%)
Duration of recording in hospital (mean ± SEM)	6.1 ± 0.4 days
Taking antiepileptic drugs (%)	10 (71%)
**Targets with leads (total across study population)**	
Number of leads (total; mean ± SEM)	204; 14.6 ± 0.6
Supplementary motor area (SMA)	8 (4%)
Amygdala	25 (12%)
Cingulate	55 (27%)
Frontal	11 (5%)
Hippocampus	25 (12%)
Insula	10 (5%)
Orbitofrontal	28 (14%)
Parietal	7 (3%)
Premotor	5 (3%)
Temporal	28 (14%)
Thalamus	2 (1%)
Number of channels (total; mean ± SEM)	2055; 146.8 ± 7.6

*SEM* standard error of the mean.

### Signal processing

High-resolution SEEG recordings sampled at 512 Hz were obtained from the Natus NeuroWorks platform, filtered with a one-pass, zero-phase, non-causal 50 Hz low-pass finite impulse response filter, and scaled to (− 1, 1). Concurrent video recordings for each patient in the monitoring unit were acquired at 480p resolution at 30 frames per second. Videos were segmented into sequential clips, converted to .tiff image files using FFmpeg, and fed into a convolutional LSTM autoencoder that was structured to have 2 convolutional layers, 3 convolutional LSTM layers, and 2 deconvolutional layers^16^. A regularity score time series was calculated for all video frames by computing the reconstruction error of each frame by summing up all pixel-wise errors, as described by Hasan et al.^15^. Signal processing was conducted using MNE 0.17.1 and SciPy Signal in Python 3.7.

### LSTM training and parameters

A self-supervised training regimen was established where each channel from the SEEG recordings and regularity score time series was divided into training and testing sequences using variable train:test splits ranging from 20:80 to 50:50. 29% of recordings in the train set had epileptiform events whereas 86% of recordings in the test set had such events (Table 2). A LSTM network with 80 hidden layers was initialized for each channel and trained on the unlabeled training sequence for up to 35 epochs (or until early stopping criteria were met) with a sequence length typically between 250,000 to 750,000 elements, which spanned anywhere from 10 to 30 min overall and either did or did not include known anomalies. To mitigate the risk of model overfitting, early stopping criteria were used while training each model. These criteria specified that training iterations must decrease the loss metric by at least 0.003 to allow additional training iterations to occur. Using a training “patience” of 5, up to 5 consecutive training iterations were allowed to occur without decreasing the loss metric by at least 0.003 before model training was stopped early. Each LSTM used a mean-squared error loss metric, an Adam optimizer, and a dropout of 0.3. Within the training sequences, 20% of the data was set aside as validation before testing. After training, the performance of each model was assessed on the unseen test sequences. The network was assessed for its ability to predict future values in real-time (Fig. 4A), compare the predictions to the actual values, and compute a smoothed error based on the difference between the actual and predicted values (Fig. 4B). LSTMs and convolutional autoencoders were implemented using TensorFlow.

**Table 2:** Neural network specifications and results.

**LSTM metrics for SEEG and videos**	
Train:test ratio (mean ± SEM)	0.41 ± 0.03
Time used to train model (mean ± SEM)	11.2 ± 1.5 min
Train recordings with events (%)	4 (29%)
Test recordings with events (%)	12 (86%)
MAPE for dynamic threshold (mean ± SEM)	0.7 ± 0.2%
MAPE for static threshold (mean ± SEM)	0.7 ± 0.1%
MAPE for crossover experiments with dynamic threshold (mean ± SEM)	2.7 ± 0.8%
MAPE for video recordings (mean ± SEM)	19.9 ± 0.8%
**SEEG static thresholding results**	
Sensitivity (mean ± SEM)	64.3 ± 13.3%
Positive predictive value (mean ± SEM)	34.4 ± 13.9%
F_1_ Score (mean ± SEM)	0.61 ± 0.12
**SEEG dynamic thresholding results**	
Sensitivity (mean ± SEM)	78.6 ± 11.4%
Positive predictive value (mean ± SEM)	89.6 ± 9.2%
F_1_ Score (mean ± SEM)	0.92 ± 0.08
**SEEG crossover results**	
Sensitivity (mean ± SEM)	83.3 ± 16.7%
Positive predictive value (mean ± SEM)	15.3 ± 10.6%
F_1_ Score (mean ± SEM)	0.54 ± 0.11
**Video anomaly detection results without SEEG**	
Sensitivity (mean ± SEM)	100.0 ± 0%
Positive predictive value (mean ± SEM)	19.1 ± 7.1%
F_1_ Score (mean ± SEM)	0.44 ± 0.07
**Combined video + SEEG anomaly detection results**	
Sensitivity (mean ± SEM)	100.0 ± 0%
Positive predictive value (mean ± SEM)	65.6 ± 9.2%
F_1_ Score (mean ± SEM)	0.65 ± 0.09

*MAPE* mean absolute percent error, *SEEG* stereoelectroencephalography, *SEM* standard error of the mean.

### Self-supervised dynamic thresholding method

A novel dynamic thresholding approach, developed by the NASA Jet Propulsion Laboratory to detect real-time anomalies in telemetry data from the Mars Rover, *Curiosity*, was adapted to our models to label anomalies based on the error values from the time series predictions^14^. In contrast to conventional static thresholds frequently used for anomaly detection (e.g. mean ± 2 standard deviations), this dynamic method uses a sliding window approach to find optimal local thresholds, such that the percent decrease in the mean and standard deviation of the smoothed error in the window is maximized if values above the set threshold are excluded. To mitigate false positives, an error pruning procedure was implemented in which the sequence of smoothed errors was incrementally stepped through, the percent decrease between time steps was computed, and steps with a percent change greater than 10% remained anomalies while steps with a change less than 10% were reclassified as normal.

### Crossover and multimodal video/SEEG detection experiments

To evaluate the patient-specific nature of the LSTM models, crossover experiments were conducted, in which models were trained on recordings from one patient and tested on another, while all other conditions remained identical to previous testing conditions, including the dynamic thresholding and error pruning methods (Fig. 2). Fourteen combinations of train and test sequences derived from the study population were randomly selected to conduct the crossover experiments.

To assess the added value of multimodal detection, the concurrent video and SEEG recordings for each patient were separately fed into the corresponding deep neural networks described previously. The resulting anomalous sequence predictions made by the self-supervised dynamically threshold in the LSTM decision function for each detection modality was then pooled before comparing the predicted anomaly times with the consensus labels of the expert panel of epileptologists. We did not encounter any disagreements among the panel regarding consensus labeling within this dataset. The results of model performance on individual patient recordings are detailed in Table 3, along with the patient’s clinical and electrophysiologic seizure manifestations.

**Table 3:** Patient-specific clinical and electrophysiologic seizure manifestations, as well as model performance on individual patient recordings.

Pt #	Clinical seizure findings	EEG seizure findings	SEEG, dynamic thresholding (PPV, sensitivity)	SEEG, static thresholding (PPV, sensitivity)	Video alone, dynamic thresholding (PPV, sensitivity)	SEEG + video, dynamic thresholding (PPV, sensitivity)
1	Generalized tonic seizure with abduction of both arms and extensor posturing of her legs.	Generalized desynchronization of the EEG background with superimposed beta frequency activity.	92.3, 100	9.7, 100	100, 100	100, 100
2	Absence seizures with repetitive eye blinking and staring.	Generalized, repetitive, spikes and polyspikes of 2 Hz.	50.0, 100	0, 0	50.0, 100	50.0, 100
3	Bilateral motor manifestations involving extension of both arms and legs.	Sentinal spike in the left amygdala followed by a slow buildup of rhythmic theta. Activity spreads to left medial temporal, parietal, and insular regions. Semi-rhythmic theta with admixed spikes in left anterior and medial cingulate.	100, 100	27.7, 100	25.0, 100	88.0, 100
4	Ictal cry with head movements and bilateral clonic body movements obscured by blankets. Arms are held in dystonic posture bilaterally with forceful jerking movements superimposed.	Starts as low amplitude beta activity in the left hippocampus with spread to left amygdala and left medial temporal lobe. Evolves to high amplitude spiky alpha and spiky theta activities. Later spread to the medial olfactory cortex.	100, 100	0, 0	25.0, 100	25.0, 100
5	Oral and head movements with vocalizations and bilateral extremity flexion. Later progresses to tonic–clonic.	Continuous atypical, generalized spike-and-wave discharges at 4 Hz in bilateral frontal, cingulate, and hippocampal regions. Subsequent burst of spike and wave activity.	100, 100	100, 100	20.0, 100	90.7, 100
6	Notable eye movement, vocalization, and some bilateral extremity movements. Eventually tonic–clonic.	Atypical, generalized spike-and-wave discharges at 4 Hz in bilateral frontal and cingulate regions followed by rhythmic spiking diffusely.	100, 100	No events detected	20.0, 100	33.3, 100
7	Multiple subclinical seizures. Clinically, all seizures are hypermotor, and begin with a rapid movement in the hands.	Slightly different onsets but nearly always maximal involvement in left lateral temporal. Begins with spike and wave, or gamma/beta activity there. Often has several minutes of very subtle epileptic spasms with diffuse slow waves in left lateral temporal.	100, 100	100, 100	20.0, 100	60.0, 100
8	Versive head movements with right arm flexion and extension, followed by tonic–clonic movements of both arms.	Rhythmic fast activity in right medial cingulate and temporal areas. Sharply contoured theta develops in left hippocampus, which evolves to spike and slow wave morphology and spreads to bilateral medial cingulate and left temporal areas.	100, 100	100, 100	25.0, 100	40.0, 100
9	Oral movements with ictal cry and right facial contraction. Later generalized clonic jerking and posturing before generalized tonic–clonic seizures.	Desynchronization with superimposed low voltage fast beta/gamma activity over left medial cingulate. Later, ictal discharge of repetitive spikes become wide spread, involving cingulate, temporal, and amygdala areas bilaterally.	No events detected	No events detected	16.7, 100	16.7, 100
10	Right arm movements with subtle leg movements. Some head movement with eyes looking up and left. Later, jerking movements, vocalization, and tonic posturing.	Rhythmic alpha activity in right amygdala and hippocampus that slows to the theta range.	No events detected	No events detected	16.7, 100	16.7, 100
11	Lower extremity bicycling movements under bed sheets.	High amplitude right hippocampal activity. Several seconds into the seizure, there is spread to right insula, cingulate, and temporal regions.	100, 100	59.0, 100	33.3, 100	50.0, 100
12	Motionless at onset. At times will look around and turn head slowly left.	Subtle low amplitude gamma buzz at left SMA. Some rhythmic beta in left premotor region.	100, 100	40.0, 100	40.0, 100	70.0, 100
13	Subtle bilateral automatisms in hands. Will then raise left hand with some tremulous movements. Later has rapid eye blinking and smile.	Right premotor area becomes rhythmic near onset but bilateral activity seen. Activity builds in amplitude and then slows to delta with admixed spikes/gamma. Some spread to left SMA.	16.7, 100	20.0, 100	50.0, 100	90.0. 100
14	Brief oral automatisms with faint vocalization. Right hand clenched into fist. Subtle clonic jerking of the right hand and arm.	Onset of repetitive spikes of 1 Hz at left hippocampus and amygdala. Spikes increase in frequency to 2–3 Hz and evolve into alpha frequency discharge. Ictal discharge spreads to left cingulate, olfactory, and temporal areas.	No events detected	No events detected	6.3, 100	6.25, 100

*EEG* electroencephalographic, *Pt* patient, *SEEG* stereoelectroencephalography, *SMA* supplementary motor area, *SEM* standard error of the mean.

### Metrics for assessing signal reconstruction quality

We assessed the models for their ability to capture the underlying signal itself using standard time series metric of mean absolute percentage error (MAPE), representing each recording channel that was reconstructed by the LSTM for each patient. The MAPEs ranged from 0.15–1.57% for each patient (average: 0.75%; Table 2), suggesting generally excellent reconstruction of the SEEG signal by the LSTM. Video regularity score signals were noisier due to diverse events occurring during recording, leading to higher MAPEs (average: 19.95%; Table 2).

### Statistics

For continuous variables in this study, the Kolmogorov–Smirnov test was first used to test for a normal distribution. Given the lack of a normal distribution in the data of this study, continuous variables were compared using the Wilcoxon–Mann–Whitney test. A threshold of p < 0.05 with two-tailed testing was used to determine statistical significance. Statistics were conducted using Prism 7.

## Data Availability

Data from this study are available upon reasonable request. In accordance with institutional policy for data protection, a Data Transfer Agreement must be completed between Mount Sinai and the requesting institution.

## Code availability

Code for the pre-processing and analysis pipelines in this study are available at: https://github.com/aisinai.
